# A Report of a Rare Case of Vagococcus fluvialis Isolated From Urine: Clinical Significance of Vagococcus Species With an Update of the Available Literature

**DOI:** 10.7759/cureus.71620

**Published:** 2024-10-16

**Authors:** Praveen R Shahapur, Roopa P Shahapur, Shreya Veggalam, Venkataramana Kandi

**Affiliations:** 1 Microbiology, Shri B M Patil Medical College Hospital and Research Centre, Bijapur Lingayat District Educational (BLDE) (Deemed to be University), Vijayapura, IND; 2 Dentistry, Shri B M Patil Medical College Hospital and Research Centre, Bijapur Lingayat District Educational (BLDE) (Deemed to be University), Vijayapura, IND; 3 Medicine, Prathima Institute of Medical Sciences, Karimnagar, IND; 4 Clinical Microbiology, Prathima Institute of Medical Sciences, Karimnagar, IND

**Keywords:** enterococcus, pan-drug resistant, urinary tract infection, vagococcus fluvialis, vagococcus species

## Abstract

*Vagococcus*species (spp.) are gram-positive cocci that are rarely reported in humans. These bacteria share physiological, cultural, and biochemical properties with *Enterococcus* spp. Recently, they have garnered attention as potential opportunistic pathogens capable of causing infections in individuals with predisposing factors and comorbidities. Human infections are sporadic, with only a few cases reported worldwide. A multidisciplinary microbiological approach is essential for the successful identification of these organisms. Antibiotic sensitivity is critical for effective treatment, especially considering that *Vagococcus* spp. not only were recently discovered but are also developing resistance to several antibiotics, as confirmed by the available literature, including the present case where the organism was found to be pan-drug resistant. Clinicians should be aware of these bacteria and consider them as emerging opportunistic pathogens. We report the case of a 56-year-old male with grade III hydronephrosis, urolithiasis, and a UTI, in which*Vagococcus fluvialis* was isolated from the patient’s urine specimen.

## Introduction

The Enterococcaceae family comprises a large group of gram-positive, spherical-shaped bacteria. This family includes several genera such as *Bavariicoccus*, *Catellicoccus*, *Enterococcus (E)*, *Melissococcus*, *Pilibacter*, *Tetragenococcus*, and *Vagococcus (V) *[1]. Approximately 25 identified species of *Vagococcus *exist, including *Vagococcus acidifermentans (2011)*, *Vagococcus allomyrinae*, *Vagococcus bubulae*, *Vagococcus carniphilus*, *Vagococcus coleopterorum*, *Vagococcus elongatus*, *Vagococcus entomophilus*, *Vagococcus fessus*, and *Vagococcus fluvialis*, among others [2].

Vagococci are motile cocci that resemble lactococci and were previously referred to as lactic streptococci. These bacteria share morphological, physiological, and some biochemical properties with the more common enterococcal group, such as being gram-positive cocci occurring in pairs and chains and being catalase-negative. Enterococci are well-recognized as commensals and potential pathogens in humans and animals [3,4]. However, due to their similar phenotypic characteristics, vagococci may be misidentified as enterococci when isolated from human clinical specimens [5]. The first species of *Vagococcus *was isolated from chicken feces and river water, identified as *V. fluvialis *in 1989 [6]. *Vagococcus salmoninarum *was identified in 1990 in relation to diseases affecting salmon and other fish species [7]. Since their initial identification occurred in animals, there is a potential for transmission of infections through animal-derived food sources such as chicken and fish, necessitating further research and confirmation.

The genus *Vagococcus *consists of facultatively anaerobic bacteria, similar to other bacteria responsible for human infections. Although they typically appear as cocci in pairs and chains, gram staining reveals that these bacteria may exhibit elongated cocci resembling rod-shaped bacteria, a morphology also observed in the *Enterococcus *group [2].

Among the various vagococcal species, *V. fluvialis *has frequently been isolated from human clinical specimens, causing rare opportunistic infections. *Vagococcus *spp. are primarily associated with infections in animals, including cows, horses, and pigs. Recently, human infections due to *V. fluvialis *have been on the rise. *V. fluvialis *can be found in diverse environments such as rivers, seawater, and sponges, and it is also present as a commensal organism in mammals, birds, and fish [5,8]. It has been isolated from the urine of healthy cattle and has been established as a probiotic in fish [9].

Initially, *Vagococcus *was isolated from animals. Due to their phenotypic characteristics, these bacteria were initially termed “unidentified enterococci,” as they share similarities with atypical arginine-negative enterococcal species. This represented the first documented evidence suggesting a potential human connection with *V. fluvialis *[10].

Currently, limited data is available regarding *V. fluvialis*, its clinical manifestations, and its antibiotic susceptibility testing (AST) profile. Advancements in bacterial identification, such as automated systems, offer promising alternatives to conventional microbiological identification methods and could be instrumental in detecting rare organisms like *Vagococcus *spp.

We report a case of a 56-year-old male with grade III hydronephrosis, suffering from urolithiasis and UTI, from whom *V. fluvialis *was isolated from a urine specimen identified using VITEK® (bioMérieux, Inc., Marcy-l'Étoile, France).

## Case presentation

A 56-year-old male patient presented with complaints of right flank pain and nausea for one week. He reported that the intermittent pain in the right flank had lasted about six months, accompanied by occasional vomiting and burning micturition. He had a previous history of urolithiasis and had undergone percutaneous nephrolithotomy and double J stent removal four years prior.

A comprehensive general physical examination revealed no unusual findings. On palpation of the abdomen, soft tenderness in the right lumbar region was noted. The patient’s vital signs showed no rise in body temperature (afebrile), with a normal heart rate of 82 beats per minute and blood pressure of 120/80 mmHg.

Peripheral blood analysis indicated mild leukocytosis, with a white blood cell count of 10,220 cells/mm³ (normal range: 4,000-10,000 cells/mm³). Other blood investigations showed normal random blood sugar, prothrombin time, bleeding time, and clotting time. Serum creatinine levels were mildly elevated at 1.6 mg/dL (normal range: 0.6-1.1 mg/dL). Serological testing for HIV, hepatitis B virus, and hepatitis C virus yielded negative results.

**Figure 1:**
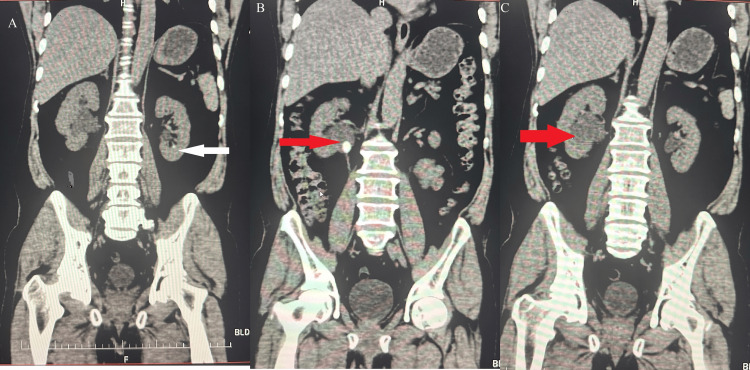
CT images showing the presence of concretions, calculi, and hydronephrosis (A) Coronal section showing a small concretion in the left kidney (white arrow). (B) Coronal section depicting a calculus in the right kidney at the PUJ (red arrow). (C) Coronal section illustrating hydronephrosis of the right kidney (red arrow). PUJ, pelvic-ureteric junction

A CT scan of the kidney, ureter, and bladder revealed multiple findings. The left kidney was normal in size (10.1 × 4.8 cm) and attenuated, with a tiny concretion of 3 mm in the lower pole calyx. The right kidney demonstrated a calculus (13 × 9 mm, 470 HU) at the pelvic-ureteric junction (PUJ) at the level of the L1-L2 intervertebral disc, causing upstream grade III hydronephrosis (Figure 1).

Mild perirenal fat stranding secondary to obstruction was noted. Degenerative changes in the spine included sacralization of the L5 vertebra and pseudoarticulation of the left transverse process of L5 with the left sacral ala, suggestive of lumbosacral transition (Castelli’s grade IIA). MRI confirmed the presence of grade III hydronephrosis.

**Figure 2:**
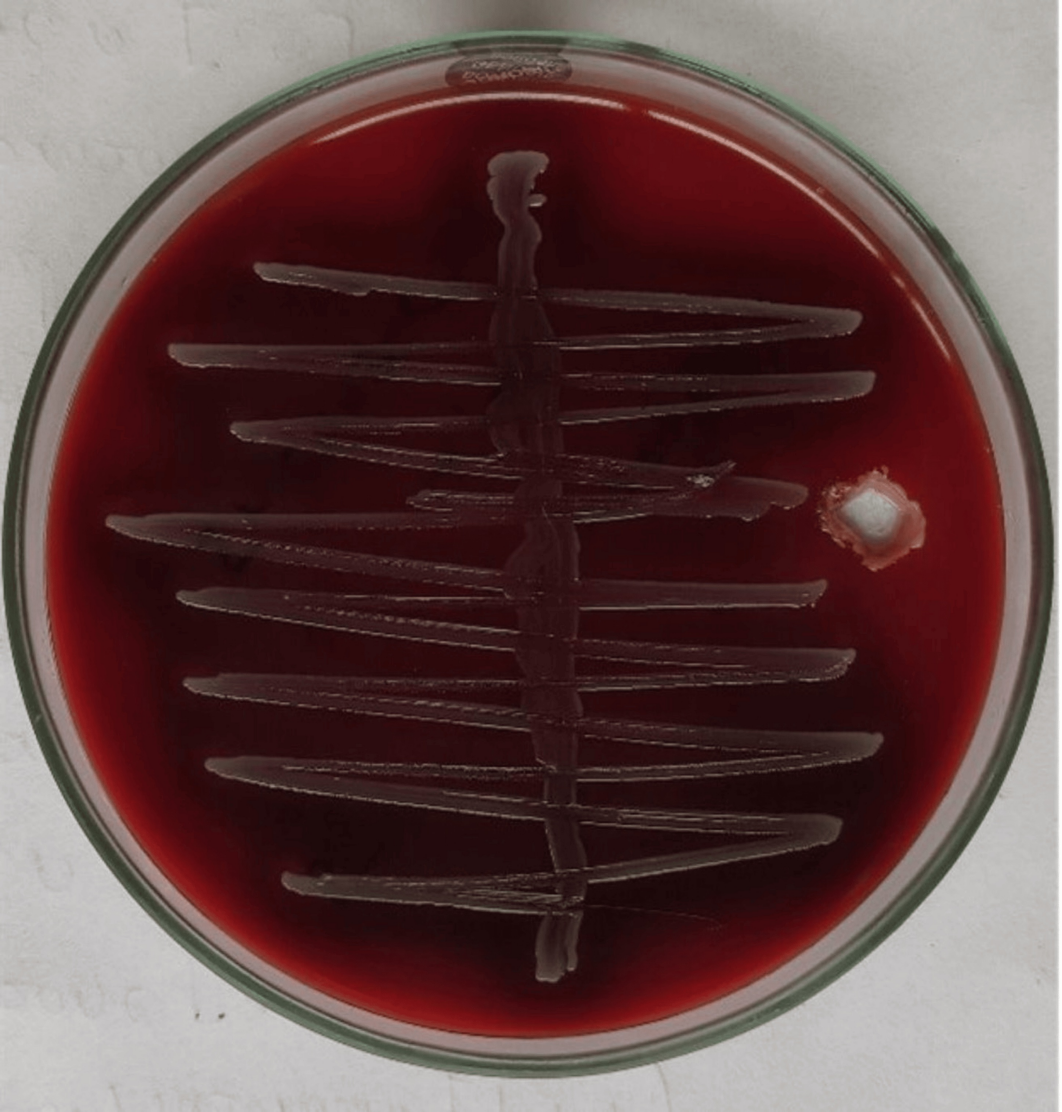
Growth of nonhemolytic colonies on blood agar

On macroscopic examination, the patient’s urine appeared yellow and cloudy. Biochemical analysis showed trace amounts of albumin. The urine culture revealed the growth of small, flat, gray-colored nonhemolytic colonies on blood agar (Figure 2).

**Figure 3:**
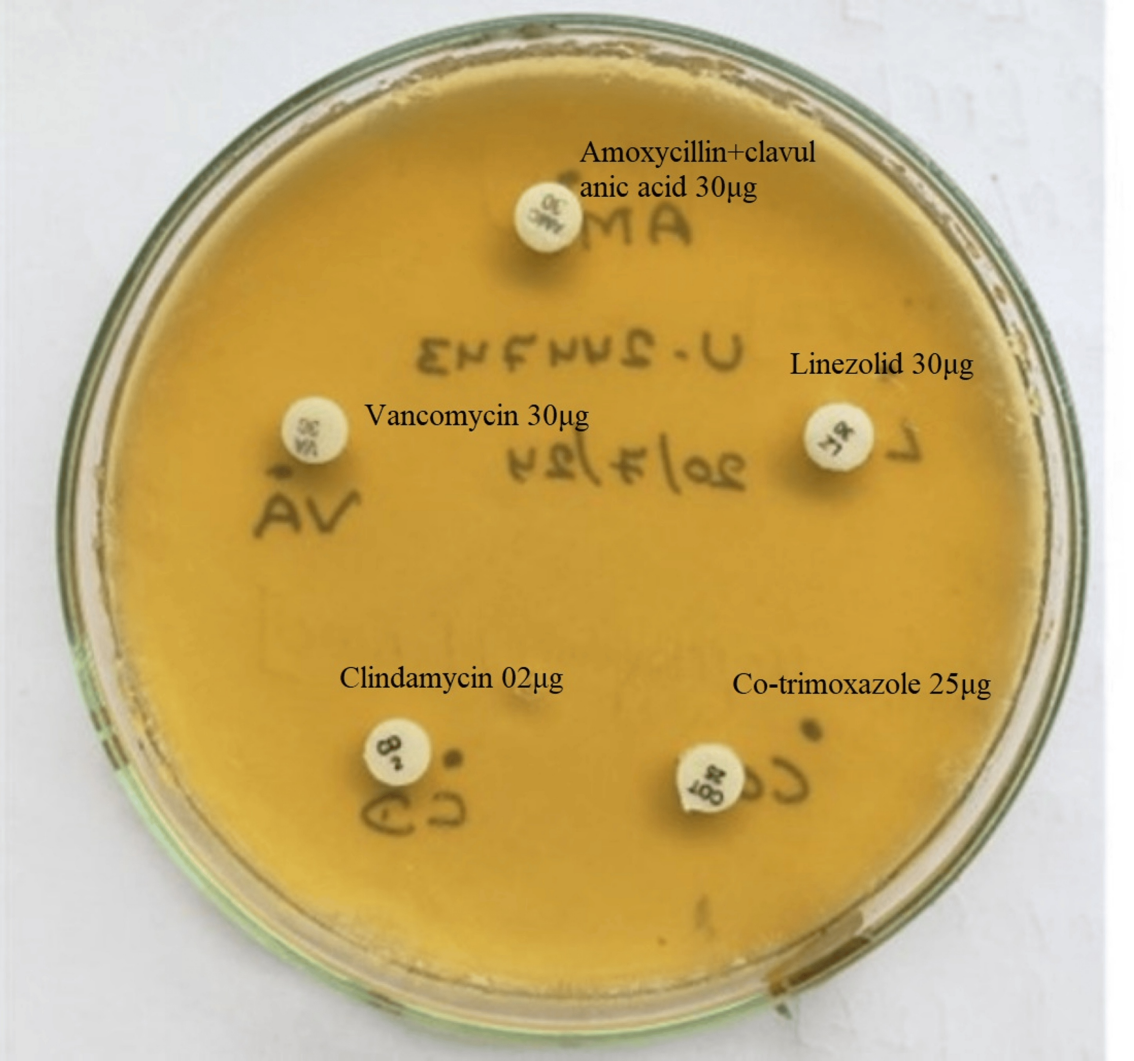
AST using the Kirby-Bauer disk diffusion method, indicating pan-drug resistance AST, antibiotic susceptibility testing

The bacterium was identified as *V. fluvialis *using the VITEK® system (bioMérieux, Inc.) with an accuracy of 89%. Antimicrobial susceptibility testing performed with the Kirby-Bauer disk diffusion method revealed that the isolated bacterium was pan-drug resistant, demonstrating resistance to vancomycin, linezolid, clindamycin, amoxicillin and clavulanic acid, trimethoprim and sulfamethoxazole (TMP-SMX), tetracycline, erythromycin, and cefoperazone, as well as piperacillin and tazobactam (Figure 3).

The patient was prescribed oral ciprofloxacin (500 milligrams twice daily) for three days, pantoprazole (40 milligrams orally once daily), and a combination of tramadol and paracetamol for two days. He was instructed to follow up after one week in the urology outpatient department and was adequately informed about the signs and symptoms requiring urgent care, including fever with chills, abdominal pain, dysuria, and hematuria. The patient’s prognosis was favorable and uneventful.

## Discussion

UTI is one of the most common complications associated with urolithiasis, which can cause obstruction at any point in the urinary tract. The presence of a calculus leads to urinary obstruction, which, over time, can progress to hydronephrosis [11]. UTIs caused by *V. fluvialis* are uncommon and rarely reported in the literature.

Management of urinary tract obstruction and UTI involves targeted antibiotic therapy based on culture and AST results. Alongside urinary drainage, interventions such as urinary bladder catheterization, percutaneous nephrostomy, and definitive surgical management are recommended [12].

The exact pathogenicity of *V. fluvialis *remains unclear. In this case, the patient has a known history of urolithiasis, having previously passed stones. CT scans revealed an obstruction at the PUJ of the right kidney, resulting in grade III hydronephrosis. UTI is a prevalent complication of urolithiasis, occurring with or without hydronephrosis, due to urine blockage within the renal system [13].

The mode of entry for the pathogen in this case could not be established. The patient was appropriately managed to alleviate the renal obstruction, thereby preventing future complications, including recurrent UTIs.

Literature review

*V. fluvialis *is an emerging pathogen associated with human infections, belonging to the Enterococcaceae family. Instances of human infection remain rare, with only a limited number of cases reported globally [14]. *V. fluvialis *closely resembles members of the *Enterococcus* and *Lactococcus *groups and is predominantly found in aquatic environments. It serves as a commensal organism in various animals, including pigs, cattle, and fish.

The first documented human infection caused by *V. fluvialis *occurred in 1989 [10]. The rising incidence of *V. fluvialis *infections in humans is likely linked to advancements in microbiologic identification technology that have enhanced the detection of this organism.

*V. fluvialis *has previously been isolated from various human clinical specimens, including blood, pus from wounds, urine, bile, peritoneal fluid, cerebrospinal fluid, and root canals in endodontic infections. The mode of transmission for this organism has yet to be established. However, in reported cases, such as a skin and soft tissue infection in a 19-year-old male resulting from a lacerated wound, transmission may occur through skin contact and penetration [14,15].

While *V. fluvialis *is detected in animals and aquatic environments, the predominant route of infection transmission remains undetermined [16]. Molecular characterization of *V. fluvialis *isolates from bovine urine, sponges, turtles, turkey feces, and chicken feces has identified several genetic factors, including mobile genetic elements such as conjugative plasmids, prophages, and insertion sequences, which potentially enhance the bacteria’s adaptability and survival in animals [8].

Advanced identification systems like matrix-assisted laser desorption ionization time-of-flight mass spectrometry (MALDI-ToF-MS) facilitate the accurate detection of rare organisms, including *V. fluvialis *[17]. Traditional identification systems may misidentify *V. fluvialis*, classifying it as common organisms such as *Enterococcus *spp. due to similar phenotypic, cultural, and biochemical characteristics [10,18,19]. Some conventional phenotypic and biochemical tests, such as growth at 45 °C, motility, the Voges-Proskauer (VP) test, arginine dehydrogenase activity, and hydrogen sulfide (H₂S) production, may be useful in distinguishing vagococci from enterococci (Table 1).

**Table 1:** Conventional laboratory test results for Vagococcus and Enterococcus species H₂S, hydrogen sulfide; PYR, pyrrolidonyl arylamidase; VP, Voges-Proskauer

Test	Vagococcus spp.	Enterococcus spp.
Catalase	Positive (100%)	Positive (100%)
Growth in 6.5% NaCl	Positive (62.5%)	Positive (100%)
Growth at 10 °C	Positive (87.5%)	Positive (100%)
Growth at 45 °C	Negative (75%)	Positive (100%)
Pyruvate utilization	Positive (50%)	Positive (100%)
Growth in tellurite medium	Positive (50%)	Positive (100%) *Enterococcus faecalis*
Motility	Motile (100%)	Nonmotile (100%)
VP test	Negative (62.5%)	Positive (100%)
PYR activity	Positive (100%)	Positive (100%)
Esculin hydrolysis	Positive (100%)	Positive (100%)
Vancomycin susceptibility	Sensitive (100%)	Variable
Acid from L-arabinose and raffinose	Positive (100%)	Negative (100%) *Enterococcus faecium*
Acid from raffinose	Positive (100%)	Negative (100%)* E. faecium*
Arginine dehydrogenase	Negative (100%)	Positive (100%)
Hippurate hydrolysis	Negative (100%)	Variable
H_2_S production	Positive (100%)	Negative (100%)

*V. fluvialis *is a recently identified emerging bacterium, with limited information available regarding its clinical manifestations, AST profile, and treatment outcomes. Currently, no standardized protocol exists for treating *V. fluvialis *infections. Evaluating the AST profile is crucial due to the limited knowledge of its susceptibility patterns to existing antibiotics. Administering appropriate antibiotics based on AST results is essential for resolving infections and preventing the future development of antibiotic resistance.

Given the rarity of this organism, clinical outcomes can vary significantly and cannot be generalized across cases. AST is integral to managing vagococcal infections, and physicians and microbiologists should consider *V. fluvialis* when evaluating atypical cases, especially those with significant comorbidities and predisposing factors.

Conventional microbiological methods often fail to identify *Vagococcus *spp., resulting in a limited number of reported human infections in the literature [14,20-26]. In most previously documented cases, identification was achieved using MALDI-ToF-MS, while only a few studies, including the present one, reported identification through the VITEK® system. Notably, in 77.79% (7/9) of the cases, patients were over 50 years old, with many being debilitated or having comorbid conditions. Details of the case studies are provided in Table 2.

**Table 2:** Global reports of Vagococcus with details of infection, diagnosis, and antibiotic sensitivity CABG, coronary artery bypass graft; MALDI-ToF-MS, matrix-assisted laser desorption ionization time-of-flight mass spectrometry; TMP-SMX, trimethoprim and sulfamethoxazole

Citation	Country	Age/sex	Diagnosis	Specimen	Clinical features	Identified by	Antibiotic sensitivity testing	Treatment and prognosis
Present case	India	56/male	Urolithiasis, grade III hydronephrosis, and UTI	Urine	Burning micturition; pain in the right flank region lasting six months with a history of occasional vomiting and	VITEK® (bioMérieux, Inc.)	Pan-drug resistant	Ciprofloxacin, a favorable outcome
Matsuo et al. (2021) [14]	Japan	74/male	An infected decubitus ulcer with foul-smelling and yellowish exudative pus on the left chest wall and abdomen, forearm, thigh, and lower leg	Blood	Muscle weakness on his left extremities, dysarthria, and altered mental status along with fever for the past four days	VITEK®2 GP ID card (bioMérieux, Inc.) and MALDI-ToF-MS	NA	Antibiotic treatment
Brunswick et al. (2024) [20]	USA	19/male	Skin and soft tissue infection due to a firework blast wound on the sole of the left foot	Pus/purulent discharge from the wound	A 7-cm laceration on the plantar aspect of his left heel, significant edema about the ankle and plantar aspect of his foot, and ecchymosis most prominent over the medial malleolus	Isolates were identified with MALDI-ToF-MS	Showed susceptibility to ampicillin, minocycline, vancomycin, and linezolid, but resistance to TMP-SMX and levofloxacin	Surgical irrigation and debridement of the wound with drain placement and the patient is started with intravenous antibiotic therapy with vancomycin and ceftriaxone. The prognosis was good.
Kitano et al. (2024) [21]	Japan	84/male	Right pyelonephritis and hydronephrosis with UTI	Pelvic aspiration of urine	A high fever that had persisted for several days with a previous history of total nephroureterectomy for left ureteral cancer, total cystectomy, and right ureterocutaneostomy	MALDI-ToF-MS; BD MALDI Biotyper Sirius system	Susceptible to penicillin, ampicillin, ampicillin/sulbactam, imipenem, and TMP-SMX	Intravenous levofloxacin then switched to oral antibiotics. The prognosis was good.
Chen et al. (2024) [22]	China	60/male	UTI with cancer of the urinary bladder	Urine	History of low-grade fever and blood in urine constantly for the past four days	VITEK®-2 Compact fully automated microbial identification and susceptibility analysis system	*Vagococcus fluvialis* is most sensitive to tigecycline, vancomycin, ciprofloxacin/levofloxacin, and linezolid; poor inhibition effect against penicillin and nitrofurantoin	Intravenous antibiotics; surgical treatment to the urinary bladder and prognosis was good.
Zhang et al. (2023) [23]	China	66/female	Chronic cholecystitis underwent cholecystectomy	Bile	Abdominal pain	VITEK®2 compact automated bacterial identification system (bioMérieux, Inc.) and MALDI-ToF MS (VITEK MS system, bioMérieux, Inc.)	AST revealed sensitivity to penicillin, erythromycin, vancomycin, linezolid, teicoplanin, and tigecycline	The patient received antibiotic treatment for a week and was discharged. The prognosis was good.
Kucuk et al. (2022) [24]	Turkey	55/male	Weakness, ascites, and hematemesis	Ascitic fluid	Cirrhosis	VITEK® 2 GP identification card	NA	The patient was started on antibiotics and the prognosis was good
Jadhav et al. (2019) [25]	India	70/male	Infective endocarditis in one year post-operative CABG	Blood	He presented with a high-grade fever of five days duration, associated with headache, myalgia, and anorexia	VITEK®2 System	Sensitive to penicillin G, gentamicin, vancomycin, and cefotaxime, but resistant to erythromycin, azithromycin, and ofloxacin	Vancomycin and gentamycin were given intravenously and the prognosis was good.
Al-Ahmad et al. (2008) [26]	Germany	NA	Infection in root-filled tooth associated with peri radicular lesions	Tooth/oral cavity	Infection in root-filled tooth associated with peri radicular lesions	MALDI-ToF-MS and 16S rRNA gene sequence analysis	NA	NA

## Conclusions

This case report, along with the existing literature, underscores* V. fluvialis *as an emerging opportunistic pathogen in humans. The phenotypic, cultural, and biochemical characteristics of *Vagococcus *spp. closely resemble those of *Enterococcus *spp., a group of commonly isolated gram-positive cocci from human clinical specimens. Given their presence in aquatic environments, individuals exposed to contaminated waters may be at increased risk for infection by these bacteria, highlighting the need for enhanced public awareness. Moreover, the isolation of *Vagococcus *spp. from animals such as cattle and fish reinforces their zoonotic potential.

A heightened level of clinical awareness, coupled with advanced identification techniques such as VITEK® and MALDI-ToF-MS, may be necessary for the accurate diagnosis of infections caused by these bacteria. Due to the limited literature available, further research is essential to elucidate the pathogenic potential of *Vagococcus *spp., along with their clinical manifestations, diagnosis, and treatment strategies. Early suspicion and identification of infections caused by *Vagococcus *spp. are crucial for effective patient management and improved clinical outcomes.
